# A New Functional MRI Approach for Investigating Modulations of Brain Oxygen Metabolism

**DOI:** 10.1371/journal.pone.0068122

**Published:** 2013-06-27

**Authors:** Valerie E. M. Griffeth, Nicholas P. Blockley, Aaron B. Simon, Richard B. Buxton

**Affiliations:** 1 Department of Bioengineering and Medical Scientist Training Program, University of California San Diego, La Jolla, California, United States of America; 2 Center for Functional Magnetic Resonance Imaging, Department of Radiology, University of California San Diego, La Jolla, California, United States of America; 3 Kavli Institute for Brain and Mind, University of California San Diego, La Jolla, California, United States of America; University of Minnesota, United States of America

## Abstract

Functional MRI (fMRI) using the blood oxygenation level dependent (BOLD) signal is a common technique in the study of brain function. The BOLD signal is sensitive to the complex interaction of physiological changes including cerebral blood flow (CBF), cerebral blood volume (CBV), and cerebral oxygen metabolism (CMRO_2_). A primary goal of quantitative fMRI methods is to combine BOLD imaging with other measurements (such as CBF measured with arterial spin labeling) to derive information about CMRO_2_. This requires an accurate mathematical model to relate the BOLD signal to the physiological and hemodynamic changes; the most commonly used of these is the Davis model. Here, we propose a new nonlinear model that is straightforward and shows heuristic value in clearly relating the BOLD signal to blood flow, blood volume and the blood flow-oxygen metabolism coupling ratio. The model was tested for accuracy against a more detailed model adapted for magnetic fields of 1.5, 3 and 7T. The mathematical form of the heuristic model suggests a new ratio method for comparing combined BOLD and CBF data from two different stimulus responses to determine whether CBF and CMRO_2_ coupling differs. The method does not require a calibration experiment or knowledge of parameter values as long as the exponential parameter describing the CBF-CBV relationship remains constant between stimuli. The method was found to work well for 1.5 and 3T but is prone to systematic error at 7T. If more specific information regarding changes in CMRO_2_ is required, then with accuracy similar to that of the Davis model, the heuristic model can be applied to calibrated BOLD data at 1.5T, 3T and 7T. Both models work well over a reasonable range of blood flow and oxygen metabolism changes but are less accurate when applied to a simulated caffeine experiment in which CBF decreases and CMRO_2_ increases.

## Introduction

Functional magnetic resonance imaging (fMRI) is commonly used to map patterns of brain activation based on blood oxygenation level dependent (BOLD) signal changes [1]. A neural stimulus rapidly causes a large increase in cerebral blood flow (CBF) that is not matched in magnitude by the change in the cerebral metabolic rate of oxygen (CMRO_2_) [2]. This mismatch, defined as the coupling ratio *n* (ΔCBF/ΔCMRO_2_), leads to an increase in blood oxygenation that in large part determines the magnitude of the BOLD response. The coupling ratio is of interest because it is not constant but rather depends on factors such as brain region, stimulus type, aging and alterations in the baseline state due to drugs such as caffeine [3]–[8]. The current paradigm for examining variability in *n* relies on the Davis model [9] to analyze combined BOLD and CBF data from two stimulus response experiments along with data from an additional calibration experiment. This is a complicated data acquisition, and the analysis is further complicated by the mathematical form of the Davis model, which tends to obscure an underlying simplicity in the relationship between BOLD, CBF and CMRO_2_ [10].

Davis and colleagues introduced this model for the BOLD effect using it as the foundation for the calibrated BOLD method, and this work remains the basis for calibrated BOLD studies today [9]. In the Davis model the BOLD signal is a nonlinear function of fractional changes in CBF and CMRO_2_, multiplied by a scaling parameter *M*. The factor *M* is a lumped parameter, which includes many variables that could scale the BOLD signal and depends on both aspects of the physiological baseline state (oxygen extraction fraction, venous blood volume, and hematocrit) and also on parameters of the data acquisition (magnetic field strength and the echo time) [10], [11]. The essence of the calibrated BOLD method is that this scaling parameter, *M*, is measured in a separate experiment. In the original Davis method and still the most commonly used approach [12]–[22], the calibration experiment to calculate *M* utilizes inhalation of a hypercapnic gas mixture to elicit BOLD and CBF responses with the assumption that CO_2_ alters CBF but not CMRO_2_ [23], [24].

However, the original derivation of the Davis model neglected intravascular signal changes and volume exchange effects associated with changes in cerebral blood volume (CBV), including changes on the arterial side that are thought to be the dominant site of CBV changes [25], [26]. Recently we developed a detailed biophysical model of the BOLD signal (DBM) [10], which includes all of these additional effects while also specifically modeling effects related to arterial, capillary and venous blood volume changes with activation. While this model is too detailed to apply routinely in the calibrated BOLD experiment because many of the physiological parameters are unknown, it provides the solid theoretical framework necessary for relating the underlying metabolic and hemodynamic changes to the measured signals.

We previously used this DBM to test the accuracy of the Davis model when applied to the analysis of calibrated BOLD data, finding that errors in the estimated CMRO_2_ change were surprisingly modest given that important components of the BOLD effect were neglected in the original derivation [10]. Effectively, the Davis model parameters were providing an approximate description of the factors that were left out, beyond the parameters’ original definition in the model, and thus complicating their interpretation in physiological terms. In addition, the choice of parameter values had a relatively weak effect on the accuracy of the estimated CMRO_2_ change, provided the model employed was used consistently to calculate both *M* from the hypercapnia experiment and also the CMRO_2_ change from the activation experiment. This observation suggested that the Davis model may be more complicated than it needs to be (despite the fact that important effects were missing from its original derivation). This prompted us to look for a model that would be both simpler mathematically and that would explicitly include the effects left out of the Davis model allowing straightforward parameter interpretation.

Here we present a new, heuristic model of the BOLD response that is a pure nonlinear function of CBF scaled by a lumped factor, which includes the CBF/CMRO_2_ coupling ratio *n*. Inspired by the simple mathematical form of this new model, we present a straightforward “*ratio method*” to test whether the blood flow-oxygen metabolism coupling ratio is the same for two stimuli using only a comparison of the BOLD and CBF response ratios. This method is independent of model parameters assuming they remain consistent across experimental states, and it does not rely on an additional calibration experiment. The reliability of the new method was tested using the DBM [10] and as a demonstration the model was used to analyze data from a recent study of visual stimulus contrast [27]. Application of this technique will expand our understanding of why the mismatch between blood flow and oxygen metabolism occurs by simplifying the approach for detecting variations in the coupling ratio for different stimuli from combined BOLD and CBF data.

When quantitative information about the CMRO_2_ change is necessary, the heuristic model can also be used in the same way as the Davis model to analyze calibrated BOLD data. To examine the accuracy of the heuristic model in this application, we again used the DBM to simulate measurements of both stimulus responses and calibration responses for different combinations of physiological states. This assessment was complementary to our previous examination of the Davis model as we again compared the results against the “true” CMRO_2_ change from the DBM [10]. This analysis demonstrates that the heuristic model has comparable accuracy to the Davis model.

## Methods and Results

### Modifications to the Detailed Biophysical Model of the BOLD Signal

The DBM includes effects of intravascular and extravascular signal changes, hematocrit (*Hct*), baseline oxygen extraction fraction (*E_0_*), blood volume fractions for different vascular compartments, changes in these volumes as CBF changes, tissue signal properties and imaging parameters [10]. In the current work, an additional feature in which the arteries are split into two compartments (large arteries - *A* and arterioles - *a*) was added in order to allow for partial oxygen desaturation of the arterioles. For simplicity, the second arteriolar compartment was modeled as equal in size to the fully saturated compartment with their sum comparable in size to previous modeling for the total arterial compartment. Desaturation occurring in the arteriolar compartment was modeled as a weighted average of arterial and venous hemoglobin saturation (Table 1, σ = 0–0.2).

**Table 1:** Input parameters to the detailed model.

Variable	Description	Best Guess	Reasonable Variation
*V_0_*	Total baseline CBV fraction	0.05	0.03–0.07
ω_A_	Arterial fraction of baseline CBV	0.1	0.05–0.15
ω_a_	Arteriolar fraction of baseline CBV	0.1	0.05–0.15
ω_v_	Venous fraction of baseline CBV	0.4	0.2–0.6
α_T_	Grubb’s constant relating total CBV to CBF	0.38	0.25–0.55
α_v_	Exponent relating venous CBV to CBF	0.2	0.1–0.38
*Hct*	Resting hematocrit	0.44	0.37–0.5
*E_0_*	Resting oxygen extraction fraction	0.4	0.3–0.5
σ	Fraction of arteriolar blood reflecting venous saturation	0.1	0–0.2
PaO_2_	Arterial partial pressure of oxygen	104 mmHg	n/a
λ	Intravascular to extravascular spin density ratio	1.15	0.9–1.3

To permit modeling of the effect of hyperoxia on the BOLD signal, we also updated the DBM to calculate compartmental oxygen saturation from oxygen partial pressures using the Severinghaus equation [28]. The arterial oxygen concentration was calculated first followed by the venous oxygen concentration using the *E_0_* and Eq (10–13) from Chiarelli et al. [29]. Venous oxygen saturation was then calculated using linear interpolation of the Severinghaus equation. Arteriolar saturation was calculated as noted in the previous paragraph and capillary saturation was calculated also using a weighted average of arteries and veins [10].

We also expanded the DBM to simulate the BOLD signal at 1.5T and 7T, since the original model was only for 3T. This required adjusting the DBM to include magnetic field specific echo time (TE) and baseline extravascular signal decay rate (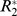) (Table 2) [30]. Intravascular signal decay rates were again determined using a quadratic model fit to data relating intravascular 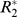 to oxygen saturation. At 1.5T hematocrit-dependent values were calculated according to Silvennoinen et al. [31]. At 7T data from Blockley et al. [32] was used to determine intravascular 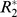 dependence on oxygen saturation independent of hematocrit. Changes in extravascular signal decay rates are linearly dependent on B_0_, which was already included in the model [1]. Calculations of oxygenation and blood volume were performed as published previously [10].

**Table 2:** Input parameter to the detailed model that are sensitive to B_0_.

Variable	Description	Best Guess			Reasonable Variation		
		1.5T	3T	7T	1.5T	3T	7T
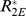	Resting extravascular rate of signal decay	11.6 s^−1^	25.1 s^−1^	35 s^−1^	9–14 s^−1^	20–30 s^−1^	28–42 s^−1^
TE	Echo time	50 ms	32 ms	25 ms	40–60 ms	20–40 ms	15–35 ms

### Simple BOLD Signal Models

The new model, as derived in Appendix S1, is:
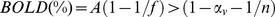
(1)


Important terms in this model include the scaling parameter (*A*), CBF in the active state normalized to its value in the baseline state (*f*), the ratio of fractional changes in CBF and CMRO_2_ (*n*), and the exponent relating the CBF change to the venous CBV change (α_v_). One additional parameter of importance is *r*, which is CMRO_2_ in the active state normalized to its value in the baseline state and is related to *n* and *f* through *n* = (*f*−1)/(*r*−1). In the following we refer to Eq. (1) as the heuristic model, because it clearly shows the basic physiological factors that affect the BOLD response: it is driven by the CBF change, but strongly modulated by both the venous CBV change and the CBF/CMRO_2_ coupling ratio. The parameter α_v_ is from the Grubb relationship, which relates the normalized venous CBV change (*v*) to *f* through the equation 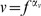. For calculations using the heuristic model, we set α_v_ = 0.2 as determined by Chen and Pike [33].

The two underlying assumptions of the heuristic model discussed and illustrated in the Appendix are: (1) The fractional BOLD signal change is directly proportional to the absolute change in total dHb content in a voxel (simulations for 1.5T, 3T and 7T are shown in Figures S1–S3); and (2) the fractional change in tissue concentration of total dHb is equal to the fractional change of venous dHb (Figure S4). Figure S5 examines the relationship between changes in CBF and CMRO_2_ in comparison to changes in both the BOLD signal and dHb content. At first glance, these assumptions appear to be too restrictive for the full complexity of the BOLD response, so we used the DBM to explore the errors in these assumptions and the ultimate effect of using the heuristic model for estimation of CMRO_2_ changes, which is discussed below.

Of note in the heuristic model is the non-linear dependence of the BOLD signal on the CBF change, which is reflected in the term incorporating *f*. This term reflects the ceiling effect on the BOLD response: very large increases in CBF will tend to produce the largest BOLD signals as 1/*f* and 1/*n* approach zero (Eq. B2). Physically this corresponds to a clearance of dHb from the vasculature. For changes in CBF approaching zero (*f* = 1), the BOLD response is a linear reflection of the fractional CMRO_2_ change as shown in Appendix S2 (Eq. B7): 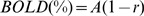.

The second term in the heuristic model relates the BOLD signal change to *n* while also incorporating the dependence of CBV on CBF. This term reflects that the largest BOLD signal will result from a large *n*, when the saturation of hemoglobin is maximized through a much lower oxygen metabolism change relative to the blood flow change (e.g. hypercapnia) [24], [34], [35]. This term also reflects that smaller changes in venous CBV relative to CBF (smaller α_v_) will lead to larger BOLD signal changes. The physical interpretation of this is that a smaller increase in dHb containing blood volume leads to a larger BOLD signal, because any increase in volume will increase the dHb content of a voxel in opposition to the oxygen extraction fraction decrease, which dominates the BOLD signal change.

For comparison, the Davis model expressed in the same terms is:
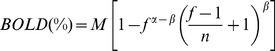
(2)


The Davis model has two parameters, α and β, and the original values for these parameters as applied to 1.5T BOLD data were α = 0.38 and β = 1.5 [9]. In this analysis, we set α = 0.2, consistent with recent data indicating that most blood volume change occurs in the arterioles [25], [26], [33]. As originally derived in the Davis model, β relates blood oxygenation to transverse relaxivity and is dependent on magnetic field strength (B_0_). Recent studies based on previous modeling of this relationship have proposed adjusting β to reflect this B_0_ dependence using the following values: β = 1.5 at 1.5T, β = 1.3 at 3T and β = 1 at 7T [9], [36], [37]. We refer to the Davis model with these parameter values as the B_0_-adjusted Davis models (e.g. the *1.5T-adjusted Davis model*).

We have also proposed previously treating α and β as free parameters in the Davis model, and designate the Davis model using these parameters as the B_0_-“free parameter” Davis models [10]. This approach attempts to provide the best fit to the surface of BOLD change as a function of CBF and CMRO_2_ change using the mathematical form of the Davis model, but divorcing the parameters α and β from their original physical definitions. The process of fitting these parameters involves assuming our best guess of the physiology (Tables 1 and 2) in order to simulate the BOLD signal for CBF changes between −40% and 80% and CMRO_2_ changes between −20% and 40%. We then normalized both the Davis model and the simulated data using an idealized hypercapnia simulation (ΔCBF = 60% and ΔCMRO_2_ = 0%). This removes the scaling parameter, *M*, from the equation leaving only α and β to be fitted. We discuss the impact of these parameters in a later section while listing their values here: α = 0.1 and β = 1 at 1.5T, α = 0.13 and β = 0.92 at 3T, and α = 0.3 and β = 1.2 at 7T. These values are perhaps counterintuitive, but when treating α and β as free-parameters they lose their physiological meaning and instead simply provide the best fit of the model to the simulated data given the physiological assumptions. In other words our values for α should not be used as an indication of the relationship between CBV and CBF, and our values for β should not be used to describe the relationship between the magnetic susceptibility due to deoxyhemoglobin and 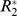. In addition to the Davis model parameter set noted above, we also examined the impact of fixing β = 1, which makes the form of the Davis model more analogous to that of the heuristic model.

### The Ratio Method

The form of the heuristic model suggests a new method for analyzing combined BOLD-CBF data independent of the scaling parameter in order to determine whether *n* changes for responses to different stimuli from the same baseline state without requiring a calibration experiment. Because the flow response term is separate from the coupling ratio term, we can use Eq. (1) to directly compare whether two stimulus responses have the same flow-metabolism coupling. Denoting one stimulus as a reference (“ref”) and the comparison stimulus as “x”, we first create a null hypothesis that *n* is the same for the two stimuli (*n*_x_ = *n*_ref_). Taking the ratio of Eq. (1) for the two stimulus responses makes a specific prediction for a nonlinear combination of measured BOLD and CBF responses that is independent of the model parameter values:
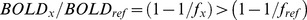
(3)


This method assumes that both *A* and α_v_ remain constant between the two stimulus responses. Under these conditions the exact values of *A* and α_v_ are not needed because this ratio is independent of the model parameters. Differences in *n* can then easily be detected using a sign rank test or similar statistical analysis comparing the measured BOLD ratio with the ratio predicted by the non-linear CBF terms for equal values of *n* in Eq (3).

**Figure 1:**
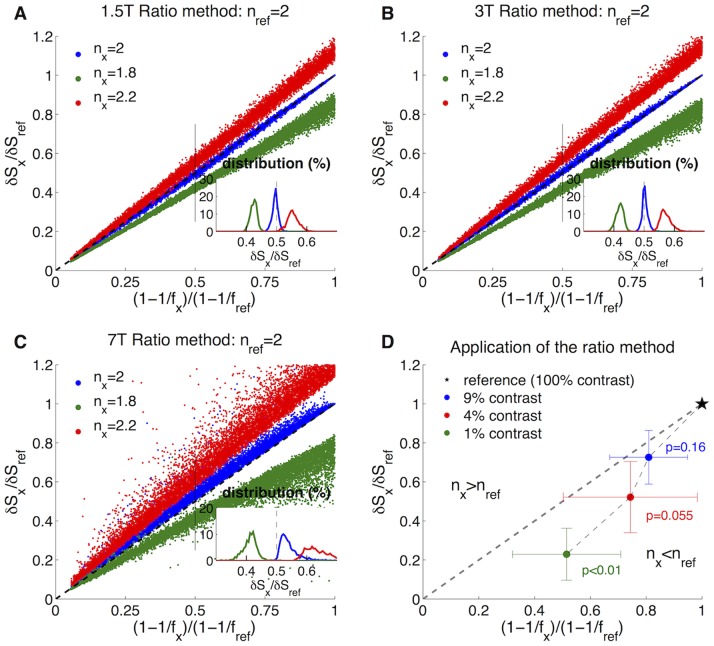
The ratio method for analysis of combined BOLD (δS) and CBF data. The DBM was used to simulate BOLD data from changes in CBF and set values of *n*. 10,000 simulations were performed using the ranges for the model inputs noted in Tables 1 and 2. The data was compared to a reference of *n_ref_* = 2. Inset histograms show the distribution of δS ratios for a non-linear CBF ratio of 0.5. At 1.5T (A) and 3T (B), the ratio method separates the data well while predicting *n_x_* = *n_ref_* data will fall along the line of identity. At 7T (C), the ratio method does not perform as well, particularly for *n_x_* = *n_ref_* for which the data deviates from the line of identity. (D) Application of the ratio method to data examining the effect of visual stimulus contrast on the coupling of CBF and CMRO_2_ in 9 subjects. 100% contrast flickering checkerboard was used as the reference with results showing that 1% contrast has a significantly lower *n*.

To test the accuracy of this new method, we employed the DBM to simulate BOLD and CBF responses for a reasonable range of physiological and imaging parameters (Tables 1 and 2). A reference data set with *n_ref_* = 2 was produced and compared to *n*_x_ = 1.8, *n*_x_ = 2 and *n*_x_ = 2.2 at 1.5T, 3T and 7T (Fig. 1A–C). These values of *n* are typical for fMRI activation experiments [7], [9], [11], [18], [27], [38]–[40]. Data sets for each value of *n* contained 10,000 simulations. Previously published combined BOLD and CBF data associated with changes in visual stimulus contrast [27] were then examined using this method (Fig. 1D). A sign rank test was used to determine whether the flow ratio was statistically different than the BOLD ratio with results for p noted.

This approach works well for 1.5T and 3T (Fig. 1A–B) as stimulus responses with values of *n_x_* not equal to *n_ref_* are shown to have BOLD ratios that diverge from the non-linear CBF ratio. Additionally when *n_x_* = *n_ref_*, the BOLD ratios are shown to be approximately equal to the non-linear CBF ratios reflected in the blue dots falling along the dashed line of identity. This is most apparent on the inset histograms taken from additional simulations for which the non-linear CBF ratio was fixed to 0.5: at 1.5T there is a very small tendency to underestimate the BOLD ratio when *n_x_* = *n_ref_*, but there is good separation between the data otherwise (Fig. 1A). Similarly at 3T the blue dots representing *n_x_* = *n_ref_* fall equally on either side of 0.5 and are separated from the data representing both *n_x_*>*n_ref_* and *n_x_*<*n_ref_* (Fig. 1B). In certain cases, this method can also be used to make inferences about changes in CMRO_2_: when *n_x_*>*n_ref_* but ΔCBF*_x_*<ΔCBF*_ref_*, then ΔCMRO_2,*x*_ must also be less than ΔCMRO_2,*ref*_. Similarly when *n_x_*<*n_ref_* but ΔCBF*_x_*>ΔCBF*_ref_*, then ΔCMRO_2,*x*_ must also be more than ΔCMRO_2,*ref*_. Note that when 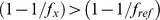 then ΔCBF*_x_*>ΔCBF*_ref_*.

This approach is less reliable at 7T where the BOLD signal is more sensitive to changes in dHb (Fig. 1C). Specifically, the ratio method fails by predicting a difference in *n_x_* from *n_ref_* when no difference exists as reflected in the blue dots deviating from the line of identify. In the case that ΔCBF*_x_* is less than ΔCBF*_ref_*, there is a tendency for this method to predict an increase in *n_x_* relative to *n_ref_*, and when ΔCBF*_x_* is greater than ΔCBF*_ref_*, there is a tendency for this method to predict a decrease in *n_x_* relative to *n_ref_*. From the inset histogram, this deviation of the data from the predicted BOLD ratio of 0.5 designated by the black bar is clearly apparent (Fig. 1C).

We also tested whether the coupling of CBF and CMRO_2_ impacts the effectiveness of the ratio method. As suggested by the form of the heuristic model, the ratio method is most sensitive to 1/*n*. By testing different values of *n*, we found that for positive coupling of CBF and CMRO_2_ the ratio method is most effective when differences in 1/*n* are greater than 0.05. For *n* = 2, this corresponds to *n* = 1.8 or *n* = 2.2. For *n* = 4, this corresponds to *n* = 3.3 or *n* = 5 (Fig. S6). We examined a wide range of both positive and negative values of *n*, and included in Figure S6 *n* = −1 corresponding to a decrease in CBF and an increase in CMRO_2_. A general pattern emerged from simulations across a broad range of coupling parameter values showing that the ratio method breaks down close to a coupling of *n* = 1.3, which is frequently associated with the null point of the BOLD signal (data not shown). Specifically at 1.5T and 3T, the ratio method appears to fail for 0.75<*n*<1.5. The limits at 7T extend somewhat higher such that the model fails for 0.75<*n*<2.25. In addition to this limitation on the range of *n* that can be examined, these tests also revealed a systematic bias in the predicted BOLD signal ratio. For positive *n*, any difference between the BOLD ratio and non-linear CBF ratio less than 0.02 should be viewed with caution and outside the ability of the ratio method to discriminate. For example, if the non-linear CBF ratio predicts a BOLD ratio of 0.5, any BOLD ratio between 0.48 and 0.52 should be considered to have the same *n*. For negative *n*, this difference is 0.04. These biases are likely due to error inherent to the use of the relatively simple heuristic model to describe the full complexity of the BOLD signal.

Having confirmed the accuracy of the ratio method for simulated data at 3T, we applied this approach to a study of 9 subjects comparing different levels of visual stimulus contrast [27]. Consistent with the results from the previous analysis using the Davis model, we found that the response to 1% contrast has a lower *n* than the response to 100% contrast (p<0.01) (Fig. 1D). The BOLD ratios at 4% and 9% contrast also fall below the prediction by the CBF ratios, but the results do not reach statistical significance. Assuming *n_ref_* = 2.3 at 100% contrast consistent with a previous calibrated-BOLD study [22], these ratio differences translate to *n* values of 1.66, 2.14 and 2.25 (with associated Cohen-*d* statistics of 0.6, 1.04 and 1.71) respectively.

### Simulating the Calibrated-BOLD Experiment

Next we simulated a calibrated-BOLD experiment to compare the heuristic model to the B_0_-adjusted Davis model [9] for accuracy in determining the CMRO_2_ change at 3 magnetic field strengths. To examine the effects of various parameters on calculations of the CMRO_2_ change, we used two ranges for *n* (*n* = 2 and *n* = −1). Activation studies typically show increases in both CBF and CMRO_2_ with *n* about equal to 2 [18], [22], [40]. In contrast, we found that caffeine as a stimulus decreased CBF and increased CMRO_2_, with *n* about equal to −1 [7]. To determine the effectiveness of the simple models, this comparison required three steps: (1) using the detailed model to simulate the hypercapnia response assuming ΔCBF = 60% and ΔCMRO_2_ = 0%, (2) using the detailed model to simulate the stimulus response with *n* = 2 (%ΔCBF = 50% and %ΔCMRO_2_ = 25%) or the caffeine response with *n* = −1 (%ΔCBF = −25% and %ΔCMRO_2_ = 25%), and (3) using the B_0_-adjusted Davis model and the heuristic model to analyze this data in order to calculate the CMRO_2_ change in response to either the simulated stimulus or caffeine experiments. Inputs to the detailed model were varied individually over the ranges specified in Tables 1 and 2 to determine the effect on ΔCMRO_2_ calculations. Parameters other than the one specified were kept constant at the best guess values.

**Figure 2:**
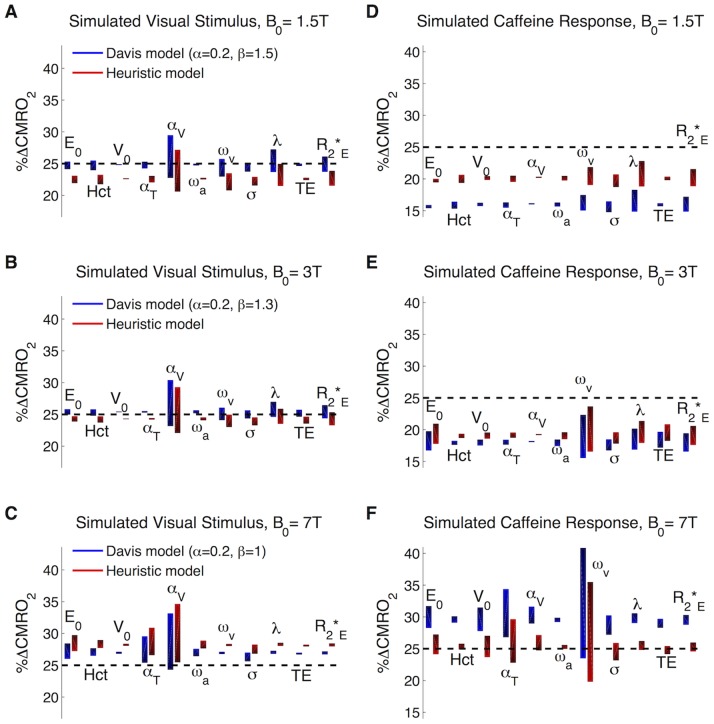
Heuristic vs. B_0_-adjusted Davis model applied to the calibrated BOLD experiment: Estimating %ΔCMRO_2_ calculation bias due to variability in physiological parameters. Eleven input parameters to the detailed model were varied around reasonable values as defined in Tables 1 and 2 while other parameters were held constant at the best guess of physiology. The activation and ideal hypercapnia experiments were simulated for each of these physiological states at (A,B) 1.5T, (C,D) 3T and (E,F) 7T. The true CMRO_2_ change with activation is shown as a dashed line, while the bars showing the range of calculated values is shaded from dark to light for increasing values of the associated physiological parameter. Davis model parameters α and β were adjusted for B_0_ as noted in the figure. In the heuristic model, α_v_ = 0.2 across all B_0_. (A,C,E) Accuracy of the models at *n* = 2 (%ΔCBF = 50%) and variable B_0_. (B,D,F) Accuracy of the models at *n* = −1 (%ΔCBF = −25%) and variable B_0_. Note values for α and β in the Davis model are consistent for each B_0_.

Figure 2 presents deviations from the DBM simulated CMRO_2_ response when using the simple models at 1.5T, 3T and 7T. These results demonstrate that %ΔCMRO_2_ calculated using the heuristic model is consistent with %ΔCMRO_2_ produced by the Davis model. It shows that even for variation in multiple physiological inputs to the DBM (Tables 1 and 2), the heuristic model with α_v_ = 0.2 [33] predicts changes in CMRO_2_ comparable to predictions by the B_0_-adjusted Davis model at 1.5T, 3T and 7T. These simple models are both quite accurate at the typical coupling ratio of *n* = 2, and at 3T there is a small underestimation bias of −6.4% error by the heuristic model compared to −2.1% for the Davis model (Fig. 2B). Both models are most sensitive to differences in α_v_, reflecting the impact of ΔCBF and venous ΔCBV coupling. If α_v_ is allowed to vary across a range of 0.1–0.3 within the DBM while the assumptions about α_v_ in the heuristic and Davis models are kept constant, then the 3T-adjusted Davis model will predict %ΔCMRO_2_ between 22.2% and 29.6% (for a true value of 25%, with a maximum error of 18.4% of that 25% change in CMRO_2_) while the heuristic model predicts %ΔCMRO_2_ between 21.5% and 28.5% (maximum error of ±14.0%). These results are consistent with the pattern found previously using slightly different values for α and β in the Davis model [10]. At 1.5T the same pattern of underestimating the CMRO_2_ change at *n* = 2 was found: for the 1.5T-adjusted Davis model the underestimation bias was −4.5% and for the heuristic model the bias was −13.3% (Fig. 2A). At 7T the basic models both overestimate %ΔCMRO_2_ with overall bias percent errors of 7.7% using the heuristic model and 2.9% using the Davis model (Fig. 2C). These patterns of bias due to parameter variation are consistent when values of *n* up to 6 (%ΔCBF = 60%) are examined (not shown).

These basic models are less accurate when used to analyze changes associated with caffeine consumption (Fig. 2D–F), which we modeled in the DBM as a −25% CBF decreases and 25% CMRO_2_ increase. This is a slightly extreme test case of CBF/CMRO_2_ coupling changes due to caffeine, since previous findings estimated a smaller CMRO_2_ increase for this level of CBF decrease [7]. At both 1.5T and 3T, the models systematically underestimate this simulated change in CMRO_2_. For example at 3T, the B_0_-adjusted Davis model calculates a CMRO_2_ increase of only 17.7% (error of −29.2%) while the heuristic model calculates 18.8% (error of −24.8%) (Fig. 2E). Of note at this value of flow-metabolism coupling, the Davis and heuristic models at 3T and 7T are most sensitive to variation in baseline dHb content as determined by ω_v_ and *E_0_*, and less sensitive to changes in blood flow-blood volume coupling, α_v_. At 1.5T the simple models are most sensitive to the intravascular/extravascular proton density ratio (λ) followed by tissue 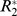 while showing less overall sensitivity to parameter variability. At 7T the heuristic model is more accurate with an error bias of −1.6% while the Davis model overestimates %ΔCMRO_2_ with an error of 15.2%. For this combination of B_0_, CBF and CMRO_2_, the magnitude of error bars is also much larger suggesting greater sensitivity to changes in dHb at 7T.

**Figure 3:**
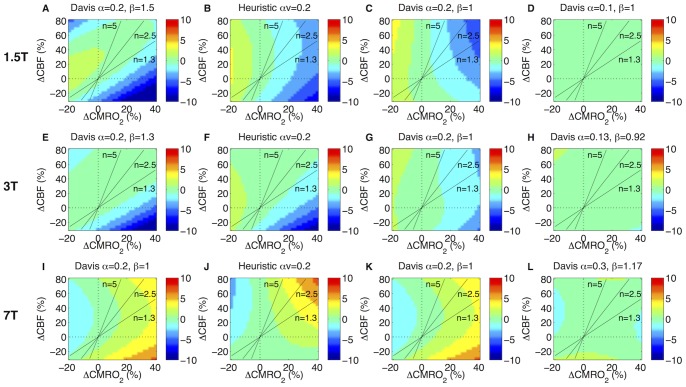
Absolute error in ΔCMRO_2­_ calculations. Simulated calibrated BOLD calculations were made for the best guess of physiology and imaging parameters noted in Tables 1 and 2. (A–D) Calculations in the absolute ΔCMRO_2­_ error are shown at 1.5T for the 1.5T-adjusted Davis model, the heuristic model, the Davis model with α = 0.2 and β = 1, and the free parameter Davis model with α and β fitted as noted. Similar calculations are shown for 3T (E–H) and 7T (I–L).

We also used this method of simulating the calibrated BOLD experiment to examine the efficacy of these simple models over a larger range of CBF and CMRO_2_ combinations while keeping other physiology constant at our best guess (Tables 1 and 2). We included in this comparison the Davis model with β = 1 (Fig. 3). As an example at 3T and for our best guess of the physiology, the simulated hypercapnia BOLD signal was 4.6% for a 60% CBF increase producing the following estimates of the scaling parameters: B_0_-adjusted, *M_HC_* = 11.4%; fixed β = 1, *M_HC_* = 14.6%; and heuristic model *A_HC_* = 15.3%. For an activation resulting in %ΔCBF = 25% and %ΔCMRO_2_ = 10%, the BOLD signal was 1.3%, and in this case the estimates of the CMRO_2_ change with activation were: 10.3% for the B_0_-adjusted Davis model, 9.3% for the fixed β = 1 Davis model, and 9.7% for the heuristic model. We also tested the impact of treating α and β as free fitting parameters within the Davis model to minimize error in CMRO_2_ calculations, and using this model ΔCMRO_2_ was estimated to equal 10.0%.

Most apparent from Figure 3 is the performance similarity of these models at different field strengths. Although subtle differences between the models exist, they all appear to function reasonably well for positive coupling of CBF and CMRO_2_ changes, particularly at 3T. Across all field strengths, the B_0_-“free parameter” Davis models perform the most consistently while the B_0_-adjusted Davis models also perform well. While we had expected the heuristic model to perform with the most similarity to the Davis model with β = 1, it in fact shares similarity to both the B_0_-adjusted and β = 1 Davis models. Notably, most of the models have difficulty correctly determining a CMRO_2_ change when it is associated with a decrease in CBF, as in changes associated with caffeine consumption. The exception to this are the free parameter Davis models and surprisingly the heuristic model at 7T (Fig. 3D, H, J, L). The drawback to the free parameter Davis model is that it requires one to discard physiological meaning for the parameters α and β. Furthermore values for α and β would need to be updated as new information affecting the DBM becomes available. Specifically α no longer corresponds to blood volume changes alone, so updating the model as new information about the true venous CBV values becomes available is more complicated. Note this is also clear from the values of α and β at 3T, which differ slightly from those published previously due to the inclusion of a desaturated arteriolar compartment here [10].

### Calibrated BOLD Analysis of Experimental Data

Using these two simple models, we examined experimental data by reanalyzing CMRO_2_ changes in response to a visual stimulus pre- and post-caffeine as well as changes due to caffeine alone [7], [22]. This data set was acquired on a GE Signa Excite 3T whole-body system using a spiral dual-echo ASL PICORE QUIPSS II pulse sequence [41]. Responses to 20 s blocks of an 8 Hz flickering checkerboard were measured pre- and post-caffeine. For complete details of the experiment see Perthen et al. [22]. Results were compared to the same data published previously so that in addition to the heuristic model we examined ΔCMRO_2_ calculations by the original Davis model (α = 0.38 and β = 1.5), 3T-adjusted Davis model (α = 0.2 and β = 1.3), and fitted free parameter Davis model (α = 0.13 and β = 0.92).

Results from this analysis using these models are shown in Table 3. The estimated values of CMRO_2_ were similar for all the models with slight systematic differences consistent with the simulations in Figures 2 and 3. The small differences in %ΔCMRO_2_ predictions reflect the similarity of these models in calculating changes in CMRO_2_ when both blood flow and metabolism increase. In contrast, the models diverge when calculating the CMRO_2_ response to caffeine alone (*n*≈−1). While the 3T free parameter model calculated a CMRO_2_ change of 21.7%, the heuristic model found 17.1%, the 3T-adjusted Davis model found 15.7%, and the original Davis model calculated 13.3% (Table 3). We anticipate that the free parameter values are most accurate in this area of CBF-CMRO_2_ and that the other models all underestimate the CMRO_2_ change for caffeine. This is consistent with our previous findings [10].

**Table 3:** Comparing ΔCMRO_2_ calculations by different models using the calibrated-BOLD approach applied to pre- and post-caffeine data.

			Heuristic^1^	B_0_-adjusted^2^	Free parameter^3^	Original^4^
	ΔBOLD (%)	ΔCBF (%)	ΔCMRO_2_ (%)	ΔCMRO_2_ (%)	ΔCMRO_2_ (%)	ΔCMRO_2_ (%)
Pre-caffeine visual response	1.2±0.09	52.1±4.25	25.9±2.3	26.9±2.3	27.1±2.4	23.4±2.0
Post-caffeine visual response	1.2±0.07	57.0±3.57	36.6±2.2	37.0±2.3	36.5±2.4	32.0±2.0
Caffeine response	−6.3±1.1	−26.9±3.48	17.1±7.3	15.7±7.3	21.7±8.5	13.3±6.4

1 Using the heuristic model proposed here with *α_v_* = 0.2,

2 Using the Davis model with α = 0.2 and β = 1.3;

3 Using the Davis model with α = 0.13 and β = 0.92;

4 Using the Davis model with α = 0.38 and β = 1.5.

### Scaling Parameters and Limits of the Davis and Heuristic Models

The above tests comparing the simple models have focused on the effects of physiological variation in properties such as CBV and hematocrit, and we can think of estimates of the scaling parameter (*M* or *A*) as the fitted value that best approximates the BOLD signal behavior over the defined physiological range. However, it is useful to also consider the limits implied by these mathematical expressions because the scaling parameter is often described in physical terms as the maximum possible BOLD signal produced when all dHb has been eliminated [9]. By this interpretation, one could in principle determine the scaling factor by extreme physiological manipulations to eliminate deoxyhemoglobin. This raises the basic question of whether these simple models remain accurate under these extreme physiological conditions. That is, is the scaling parameter in the model best thought of as an absolute physiological variable or as a fitting parameter that adjusts the mathematical form to be accurate over a normal physiological range?

To address this question we considered the limiting forms of the simple models and compared them with the limits calculated from the DBM. This is a somewhat subtle question because the elimination of dHb can be accomplished through two basic paths: a dramatic increase of CBF (perhaps augmented with hyperoxia), or a reduction of absolute CMRO_2_ to zero. We considered both scenarios with the DBM. First we modeled the elimination of dHb based on the carbogen-10 experiments by Gauthier et al. [42] allowing CBF to increase by 200%, slightly more than their finding of 160% produced using combined visual stimulus with 10% hypercapnia. We then combined this increase in CBF with an increase in arterial oxygen partial pressure (PaO_2_) up to 600 mmHg consist with about 90% inspired O_2_ [29]. We also included a simulation with ΔCBF = 100% (*f* = 2) and PaO_2_ = 390 mmHg to mimic the actual findings from the carbogen-10 experiments [42]. No literature was found on the relationship of CBV to CBF as blood flow increases beyond typical physiological measurements to provide an empirical basis for modeling such effects within the DBM, so here we kept α_T_ and α_v_ constant at 0.38 and 0.2 respectively [33], [43]. Second, to simulate oxygen metabolism cessation, the CMRO_2_ input to the DBM was simply decreased to zero without altering CBF or any other input. While not physiologically plausible, this simulation mimics complete removal of all dHb without altering CBV.

**Figure 4:**
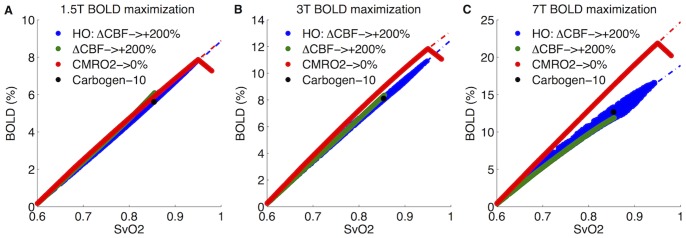
Simulating the maximum BOLD signal through dHb elimination. The maximum BOLD signal results from complete elimination of dHb, which can be accomplished by increasing CBF, decreasing CMRO_2_ and/or increasing PaO_2_. Here the BOLD signal is shown as a function of SvO_2_ at (A) 1.5T, (B) 3T, and (C) 7T. Three mechanisms of dHb reduction are included: hyperoxia combined with CBF increase (blue), CBF increase only (green) and CMRO_2_ cessation (red). Also included is a simulation for ΔCBF = 100% and PaO_2_ = 390 mmHg consistent with findings from Gauthier et al. [42].

Note that one complexity of extending the DBM to these extreme physiological cases is that we model the intravascular and extravascular susceptibility difference as being minimized at a hemoglobin saturation of 95% rather than 100% (SO_2,off_ = 95%) based on the work of [44]. Specifically, this assumes that the susceptibility of tissue is equal to the susceptibility of plasma (an assumption that needs to be tested experimentally). This results in the maximum BOLD signal occurring at a hemoglobin saturation less than 100%. Since we are after a calibration factor that reflects a constant relationship between the BOLD signal and hemoglobin saturation, we chose to extrapolate to the theoretical maximum BOLD signal at 100% SvO_2_ by projecting from the inflection point using the inverted slope of the BOLD signal for SvO_2_ greater than 95% (Fig. 4, dashed line). We modeled these mechanisms using the DBM and plotted BOLD vs. venous hemoglobin saturation (SvO_2_) in order to determine the most appropriate definition for the scaling parameters (Fig. 4).

At 1.5T the combined hyperoxia with CBF increase appears nearly identical to elimination of CMRO_2_ as both approach a limit of 8.9%. At higher magnetic field strengths, these cases diverge as the increase in CBF leads to the displacement of tissue volume for blood volume, which has a smaller contribution to the signal at higher B_0_. At 3T, elimination of CMRO_2_ results in a maximum signal of 13.2% while combined hyperoxia-CBF increase produces a signal of 12.5%. The simulated carbogen-10 experiment at 3T resulted in a signal of 8.0%, which is close to the actual finding of 7.5%. The difference between dHb elimination methods is even larger at 7T: decreasing CMRO_2_ results in a maximum signal of 24.7%, but combined hyperoxia-CBF increase produces a maximum signal of 18.9%. The difference at 7T was expected, because increased CBF leads to increased CBV replacing tissue volume without contributing to the BOLD signal at the higher magnetic field strength [45].

The limits of the Davis and heuristic model can also be examined for these two conditions with interesting differences arising. At very large values of CBF the heuristic model predicts a signal that is less than the maximum BOLD signal: *A*(1-α_v_) (Appendix S2, Eq. B2) while the Davis model predicts that the BOLD signal will simply equal *M.* At 1.5T and 3T, Figure 4 suggests this decrease is too aggressive since in the DBM simulations α_v_ was assumed to equal 0.2, but at 7T the heuristic model appears to more accurately reflect behavior at this limit.

When absolute CMRO_2_ is reduced to zero (*r* = 0), the heuristic model predicts dependence of the BOLD signal on both CBF and α_v_: 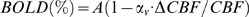 (Appendix S2, Eq. B3). When the CBF change is small as in Figure 4, this limit becomes the scaling parameter, *A*. Under the same circumstances, the Davis model reduces to *M* with no dependence on α_v_ or CBF.

These results show that the maximum BOLD signal therefore is dependent on how elimination of dHb is achieved, and for both simple models there are discrepancies between the value of the scaling parameters and the physical limits of reducing deoxyhemoglobin. For this reason, it is better to think of the scaling parameter as a fitted value that makes the equations accurate over a normal physiological range, rather than having a more absolute meaning as the maximum possible BOLD change.

### Ethics Statement

The institutional review board at the University of California San Diego approved the study of human subjects in the previously published work [22], [27], and written informed consent was obtained from all participants.

## Discussion

In this study, we revisited basic modeling of the BOLD signal and derived a new simplified model that has heuristic value in clearly showing the physiological factors that affect the BOLD signal. The heuristic model demonstrates the non-linear dependence of the BOLD signal on cerebral blood flow found in previous studies [46], [47], directly incorporates the flow-metabolism coupling parameter, *n*, and also incorporates the dependence of venous CBV on CBF through α_v_. It was inspired by work with the much more detailed model [10], which appeared to produce a very smooth BOLD dependence on CBF and CMRO_2_ suggesting that the parameters α and β of the Davis model may be over-fitting the data. The form of the heuristic model suggests a new method comparing BOLD signal ratios to non-linear ΔCBF ratios in order to determine whether flow-metabolism coupling varies with the stimulus. Using the previously developed DBM [10], we demonstated the effectiveness of the ratio method while also showing that the accuracy of the heuristic model is comparable to the Davis model when applied in the calibrated BOLD experiment.

### Ratio Method

The new approach to analyzing combined BOLD and CBF data is straightforward and relies only on measured data to determine whether *n* varies with the stimulus (i.e. different levels of visual stimulus contrast, frequencies of finger tapping, or level of drug administration). As an example, we used the method to reanalyze a previous study investigating how *n* varies with the contrast of a visual stimulus. In the original analysis the conclusion that *n* varies with stimulus contrast was based on many repeated tests using the Davis model with different values for *M*, α and β. Here using the ratio method, the same conclusion is reached in a more straightforward manner. It is not apparent from the Davis model that the comparison of the BOLD signal ratio to the non-linear CBF response ratio would work, but it is readily apparent from an examination of the heuristic model, which separates the CBF response from the coupling parameter term. An additional application of the ratio method could be in the study of brain diseases with altered vascular responses. For example in diseases resulting in reduced blood vessel compliance, increasing stimulus intensity may not result in the same increase in the CBF response seen in normal subjects resulting in a constant or decreasing *n* rather than increasing *n*.

There are two limitations of this method: both the scaling parameter and the relationship between CBF and CBV changes (α_v_) must remain constant across the comparison. The requirement on the scaling parameter to remain constant essentially limits the technique to a common region of interest and baseline state. For example, it is not possible using this method to compare flow-metabolism coupling in the visual cortex to that in the motor cortex.

An additional limitation emerges at 7T. Simulations using the DBM confirm that the approach works well for 1.5T and 3T with the non-linear CBF ratio accurately predicting the BOLD signal ratio in all cases (Fig. 1A–B), but at 7T the relationship between the ratios is not a reliable prediction of changes in *n*. Specifically if the *n*-values are in fact the same for two stimuli, the ratio method at 7T would incorrectly show that the stimulus with the stronger CBF response had a smaller value of *n*. Thus although the heuristic model works reasonably well when calculating %ΔCMRO_2_ from calibrated BOLD data at all magnetic field strengths (Fig. 2 and 3), the ratio method is unreliable at 7T. A much smaller bias is evident at 1.5T, but this deviation from identity is small as demonstrated from the inset histogram (Fig. 1A).

### Calibrated-BOLD using the Heuristic Model

As performed previously for just the Davis model, it is possible to calculate stimulus associated changes in CMRO_2_ using the heuristic model when BOLD and CBF measurements are combined with a hypercapnia calibration. To test this we simulated both an ideal hypercapnia calibration as well as an activation experiment using the DBM demonstrating that both the heuristic model and the B_0_-adjusted Davis model produce reasonable estimates of ΔCMRO_2_ (less than 15% error) for positive changes in CBF and CMRO_2_ (Fig. 2A–C). At 1.5T and 3T both models slightly underestimate changes while they overestimate changes in CMRO_2_ at 7T. As previously reported [10], the parameter having the largest impact on CMRO_2_ calculations by both models and across B_0_ is α*_v_*, emphasizing the importance of accurately determining the venous CBV-CBF relationship for future calibrated BOLD studies. In terms of the effect of other physiological parameters, an interesting standout at 1.5T is λ, which is the intravascular/extravascular proton density ratio. This alters the intravascular to extravascular signal intensity, a factor that is more important at lower B_0_ where the intravascular signal due to lower intravascular signal decay rates has a relatively greater impact than at 3T and 7T. At 7T another interesting standout is α_T_, which emphasizes the importance of total CBV changes at the higher magnetic field when deoxygenated blood generates a relatively weak signal so that increases in blood volume displace tissue without contributing to the BOLD signal leading to an overall signal decrease. Not shown here, the same pattern of error is found when using the B_0_-“free parameters” in the Davis model, which is consistent with previous findings [10].

While both models are reasonably accurate for cases in which both CBF and CMRO_2_ increase, the models are less accurate when CBF and CMRO_2_ changes are in opposition (Fig. 2D–F). Specifically for the Davis model, the B_0_-adjusted values of β underestimate ΔCMRO_2_ at 1.5T and 3T while overestimating it at 7T. The heuristic model also does not perform well at 1.5T and 3T in this region of CBF-CMRO_2_ coupling, but interestingly it is much more accurate at 7T. This is consistent with findings in Figure 3 examining a broad range of CBF-CMRO_2_ coupling for our best guess of physiology. Application of these models to experimental data showed a similar pattern of CMRO_2_ changes estimated for a visual stimulus response with the simple models in agreement with the exception of the original Davis model (α = 0.38 and β = 1.5), which estimated a smaller CMRO_2_ response (Table 3). Also consistent with the simulations, the caffeine CMRO_2_ responses calculated by the basic models were more dissimilar: the original Davis model produced the lowest estimate, the B_0_-adjusted Davis model and the heuristic model produced slightly higher estimates, and the free parameter Davis model produced the highest estimate (Table 3). The inaccuracy of the Davis model for this region of CBF and CMRO_2_ coupling has been noted previously and can be overcome by treating α and β both as free parameters in the Davis model then fitting to DBM simulations [10]. The drawback to this approach is that the parameters lose their physiological meaning and must be refitted when new information becomes available.

Examining a full complement of CBF and CMRO_2_ changes, Figure 3 also shows that fixing β = 1 decreased accuracy of the Davis model for the most common region of CBF-CMRO_2_ coupling while there was also unexpected improvement in the region of CBF decrease and CMRO_2_ increase. Although β = 1 simplifies the Davis model in line with the simplicity of the heuristic model, it is still not obvious that the ratio method would work due to the interaction of the CBF and CMRO_2_ terms.

### The Scaling Parameter and Additional Comparison of the Simple Models

When simple models of the BOLD effect are used, the physical meaning of the scaling parameter (i.e., its relationship to underlying physiological variables) can become blurred. Here we considered the question of whether the scaling parameter is literally the maximum BOLD signal change that would occur if all of the deoxyhemoglobin was removed, or whether it functions as a fitting parameter that differs based on the mathematical form of the particular simple model, adjusting each to fit the data over the normal physiological range. From Figure 4, it is apparent that the maximum BOLD signal depends on whether dHb is eliminated by increasing CBF or decreasing CMRO_2_. Additionally while the limit of the Davis model in both cases is *M*, the limit of the heuristic model is either *A* when CMRO_2_ goes to zero or *A*(1-α_v_) when CBF approaches infinity. Finally both simulations and experimental data show that hypercapnia determined values of the scaling parameter depend not only on the simple model used but also on the values of the parameters α, β and α_v_ [7], [10]. Therefore to maximize accuracy of the simple models and avoid ambiguity introduced by making the scaling parameter equivalent to the maximum BOLD signal, it is better to determine the scaling parameter from a calibration experiment, thereby providing a good fit of the simple models to the physiologically reasonable range of CBF and CMRO_2_ changes.

Our simulations provide further evidence of this: although the B_0_-adjusted Davis *M_HC_* is smaller than the heuristic model *A_HC_* at 3T, both simple models estimate CMRO_2_ changes well (Fig. 2 and 3). Furthermore the difference in *M_HC_* between the original and free parameter Davis models published previously [10] suggests strong covariance between the scaling parameter (*M*), α, and β in the Davis model. It is through the calibration process that the simple models become self-correcting, emphasizing that the value of the scaling parameter depends on the model used to calculate it rather than on the maximum BOLD response.

### Future Applications

A potentially useful feature of the heuristic model is that if the variation of *n* over time during an activation experiment is relatively small, the BOLD response becomes simply a scaled version of a pure non-linear function in CBF. In models relating the BOLD response to underlying physiology (e.g., as a component of dynamic causal modeling [46]), the ambiguities due to the baseline state and the CBF/CMRO_2_ coupling ratio are combined into a single scaling parameter, simplifying the treatment of the forward model from neural responses to measured BOLD responses.

As shown recently, the heuristic model is also useful for improving the precision of the CBF response when simultaneous measurements of BOLD and CBF are acquired [48]. By isolating dependence of the BOLD signal on the non-linear CBF response, the unknown parameters *A*, α_v_ and *n* that modulate the BOLD signal can be combined into a single factor. This property relating the underlying CBF fluctuations simply to the BOLD signal is used by the BOLD-Constrained Perfusion (BCP) method to dramatically improve the estimate of CBF fluctuations. Specifically, the heuristic model is used as a constraint in the minimization of the cost function, which incorporates the measured BOLD signal, the measured CBF signal, the true underlying BOLD and CBF signals, and noise.

### Conclusions

The heuristic model was inspired by work with the detailed BOLD model and a desire to develop a simple analysis for detecting changes in flow-metabolism coupling from combined BOLD and blood flow data. The heuristic model is advantageous over previous models, because it simplifies the dependence of the BOLD signal on blood flow and flow-metabolism coupling and in doing so suggests the ratio method for analysis of combined BOLD and CBF data. This approach works very well at 1.5T and 3T, but does not appear to work at 7T when it predicts a change in *n* when no change is present. It is remarkable to note that when applied to calibrated BOLD data the heuristic model with only one fixed parameter has accuracy similar to the Davis model with parameters adjusted for the magnetic field strength. At 1.5T, 3T and 7T, the heuristic model produces consistent results for ΔCMRO_2_ at *n* = 2, although they are slightly less accurate than the B_0_-adjusted Davis model. This small difference is balanced by greater accuracy of the heuristic model when applied to a simulated analysis of the response to caffeine particularly at 7T, which is a somewhat surprising result given the simplicity of the heuristic model.

## Supporting Information

Figure S1
**Relationship between the BOLD signal change and the total change in dHb content (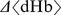) at 3T.** Scatter plots were produced by independently varying ΔCBF (−50% to 80%) and ΔCMRO_2_ (−30% to 50%) within the specified ranges. Purple curves are identical in all subplots with the exception of (D) and represent the best guess physiological case (Tables 1 and 2). (A) For the best guess of physiological parameters, the relationship between the BOLD signal and 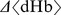 is linear, but there is a finite width to the curve. In this case, 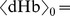
0.11 mmol of dHb per liter of tissue. For ΔBOLD between −3% and 3%, a fit to this line gives ΔBOLD(%) = −138*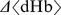. Inset is a histogram of ΔBOLD probability distribution around 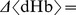
0±0.025 mg/mL (i.e., variation in the BOLD signal that could result when there is no change in net tissue dHb). (D) Allowing a wider and still reasonable distribution of physiology (Tables 1 and 2, Reasonable Variation) produced more scatter in the relationship between ΔBOLD and 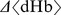. For ΔBOLD between −3% and 3%, a fit to this line gives ΔBOLD = −133*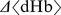. Inset is a histogram of ΔBOLD probability distribution around 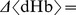
0±0.025 mg/mL. The remaining panels show how the curve changes when one of the physiological variables is altered: (B) varying baseline CBV fraction; (C) varying baseline venous and capillary CBV fractions; (E) varying the exponent relating CBF and venous CBV; (F) altering TE.(TIF)

Figure S2
**Relationship between the BOLD signal change and the total 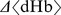 at 1.5T.** Scatter plots were produced by independently varying ΔCBF and ΔCMRO_2_ as in Figure S1. (A) For the best guess of physiology, the relationship between the BOLD signal and 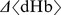 is linear, but again there is a finite width to the curve. For ΔBOLD between −3% and 3%, a fit to this line gives ΔBOLD(%) = −96*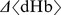. The inset is a histogram of ΔBOLD probability distribution around 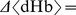
0±0.025 mg/mL is similar to that at 3T As expected, the BOLD signal shows weaker dependence on the change in dHb content than at 3T (B–F).(TIF)

Figure S3
**Relationship between the BOLD signal change and the total 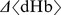 at 7T.** Scatter plots were produced by independently varying ΔCBF and ΔCMRO_2_ as in Figure S1. (A) For the best guess of physiology, the relationship between the BOLD signal and 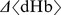 is linear with a tighter distribution than at 3T or 7T. For ΔBOLD between −3% and 3%, a fit to this data gives ΔBOLD(%) = −207*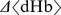. As expected, the BOLD signal shows stronger dependence on the change in dHb content than at 3T (B–F).(TIF)

Figure S4 **Relationship between the normalized venous change and normalized total change in dHb contents.** Scatter plots were produced as in Figure S1 by independently varying ΔCBF (−50% to 80%) and ΔCMRO_2_ (−30% to 50%) within the specified ranges. Purple curves are identical in all subplots with the exception of (D) and represent the best guess physiological case (Tables 1 and 2). (D) Combined variation of the parameters within the reasonable ranges (Tables 1 and 2). The only physiological variable that created a slight deviation from the identity line is the venous flow-volume relationship expressed as α_v_ (E).
(TIF)

Figure S5
**Comparing zero BOLD response to zero change in total dHb content.** This plot of the BOLD response as a function of changes in CBF and CMRO_2_ was generated using our best guess of the physiological inputs to the DBM model at 3T (Tables 1 and 2). The color scale represents the BOLD signal as a percent change. The dot-dash line represents 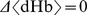 while the solid orange line represents ΔBOLD = 0%. For positive changes in CBF and CMRO_2_, 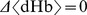 is shown to be associated with a small positive BOLD signal. This is due to the intravascular effects of dHb: although the increase in CBV and decrease in dHb concentration combine to produce no change in total dHb content and no change in the extravascular signal, the intravascular signal decay rate decreases due to the decrease in dHb concentration.(TIF)

Figure S6 **The ratio method for analysis of combined BOLD (δS) and CBF data: effects of different** ***n*** **.** The DBM was used to simulate BOLD data from changes in CBF and set values of *n*. 10,000 simulations were performed using the ranges for the model inputs noted in Tables 1 and 2. The data was compared to a reference of *n_ref_* = −1 or *n_ref_* = 4 at B_0_ = 1.5T, 3T and 7T. Inset histograms show the distribution of δS ratios for a CBF ratio of 0.5. (A,C,E) For *n_ref_* = 4 at 1.5T (A) and 3T (C), the ratio method appears to work well, although the data is slightly more difficult to distinguish, which is expected due to the decreased sensitivity of the BOLD signal to *n* at higher values of *n*. At 7T (E), the approach is again biased when *n_x_* = *n_ref_*. At all three field strengths, the ratio method separates the data well for *n_ref_* = −1, although there is bias in the *n_x_* = *n_ref_* data. (B,D,F).
(TIF)

Appendix S1 **Derivation of the new model.**
(DOC)

Appendix S2 **Limits of the simple models.**
(DOC)
