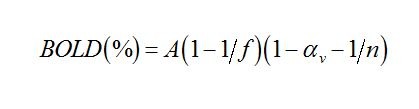# Correction: A New Functional MRI Approach for Investigating Modulations of Brain Oxygen Metabolism

**DOI:** 10.1371/annotation/3cbbc36b-12ee-4ec3-ae8e-14e8e7209018

**Published:** 2014-01-10

**Authors:** Valerie E. M. Griffeth, Nicholas P. Blockley, Aaron B. Simon, Richard B. Buxton

An error was introduced during the preparation of this article for publication. In the Methods and Results section, there is error in Equation 1. Please view the complete, correct equation here: