# Supplementary figures and images for: A New Functional MRI Approach for Investigating Modulations of Brain Oxygen Metabolism
Source: PLoS One. 2013 Jun 27;8(6):e68122. doi: 10.1371/journal.pone.0068122 (PMC3694916; doi:10.1371/journal.pone.0068122)

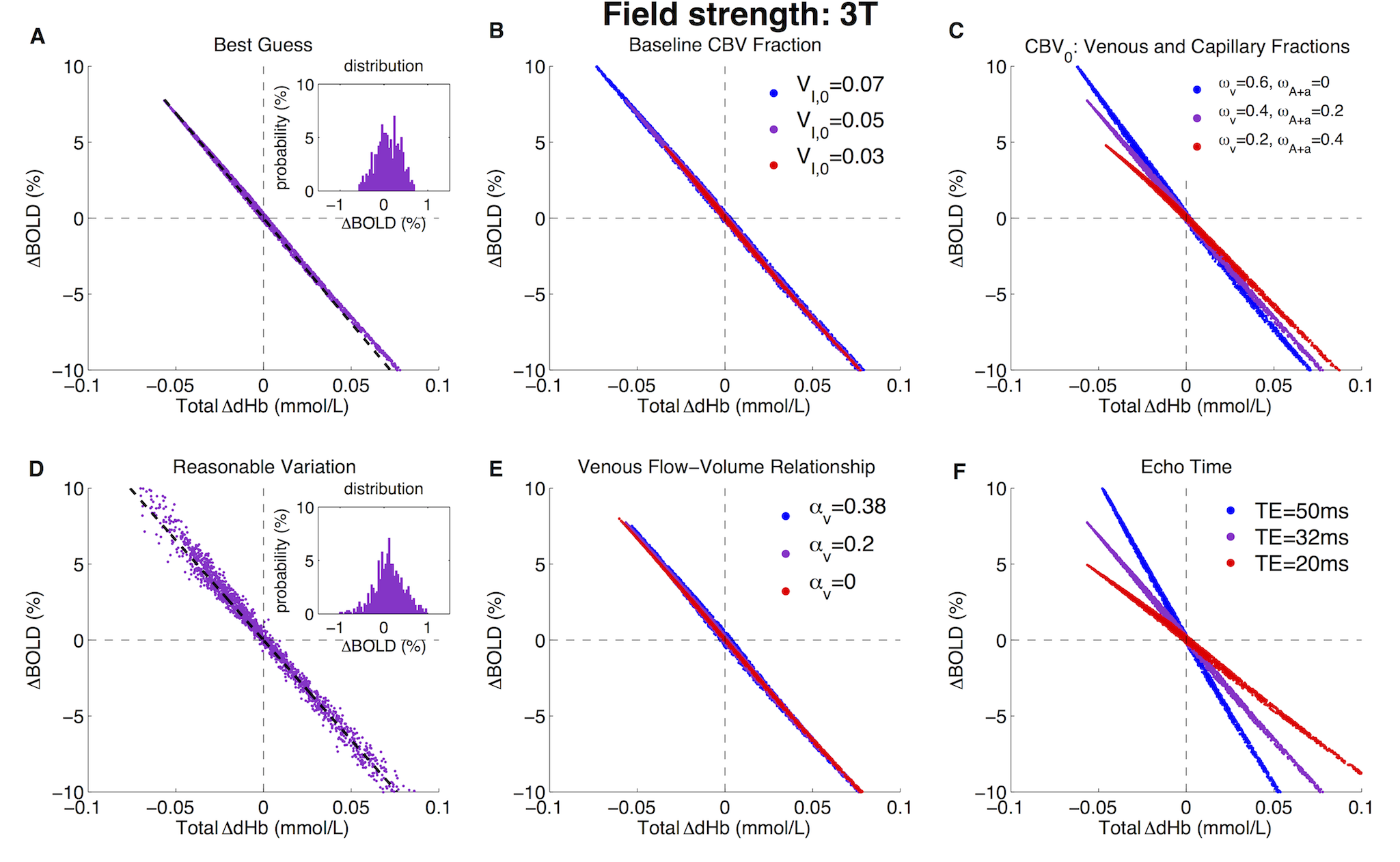

Supplement: Figure S1 — Relationship between the BOLD signal change and the total change in dHb content () at 3T. Scatter plots were produced by independently varying ΔCBF (−50% to 80%) and ΔCMRO2 (−30% to 50%) within the specified ranges. Purple curves are identical in all subplots with the exception of (D) and represent the best guess physiological case (Tables 1 and 2). (A) For the best guess of physiological parameters, the relationship between the BOLD signal and is linear, but there is a finite width to the curve. In this case, 0.11 mmol of dHb per liter of tissue. For ΔBOLD between −3% and 3%, a fit to this line gives ΔBOLD(%) = −138*. Inset is a histogram of ΔBOLD probability distribution around 0±0.025 mg/mL (i.e., variation in the BOLD signal that could result when there is no change in net tissue dHb). (D) Allowing a wider and still reasonable distribution of physiology (Tables 1 and 2, Reasonable Variation) produced more scatter in the relationship between ΔBOLD and . For ΔBOLD between −3% and 3%, a fit to this line gives ΔBOLD = −133*. Inset is a histogram of ΔBOLD probability distribution around 0±0.025 mg/mL. The remaining panels show how the curve changes when one of the physiological variables is altered: (B) varying baseline CBV fraction; (C) varying baseline venous and capillary CBV fractions; (E) varying the exponent relating CBF and venous CBV; (F) altering TE. (TIF) [file pone.0068122.s001.tif]

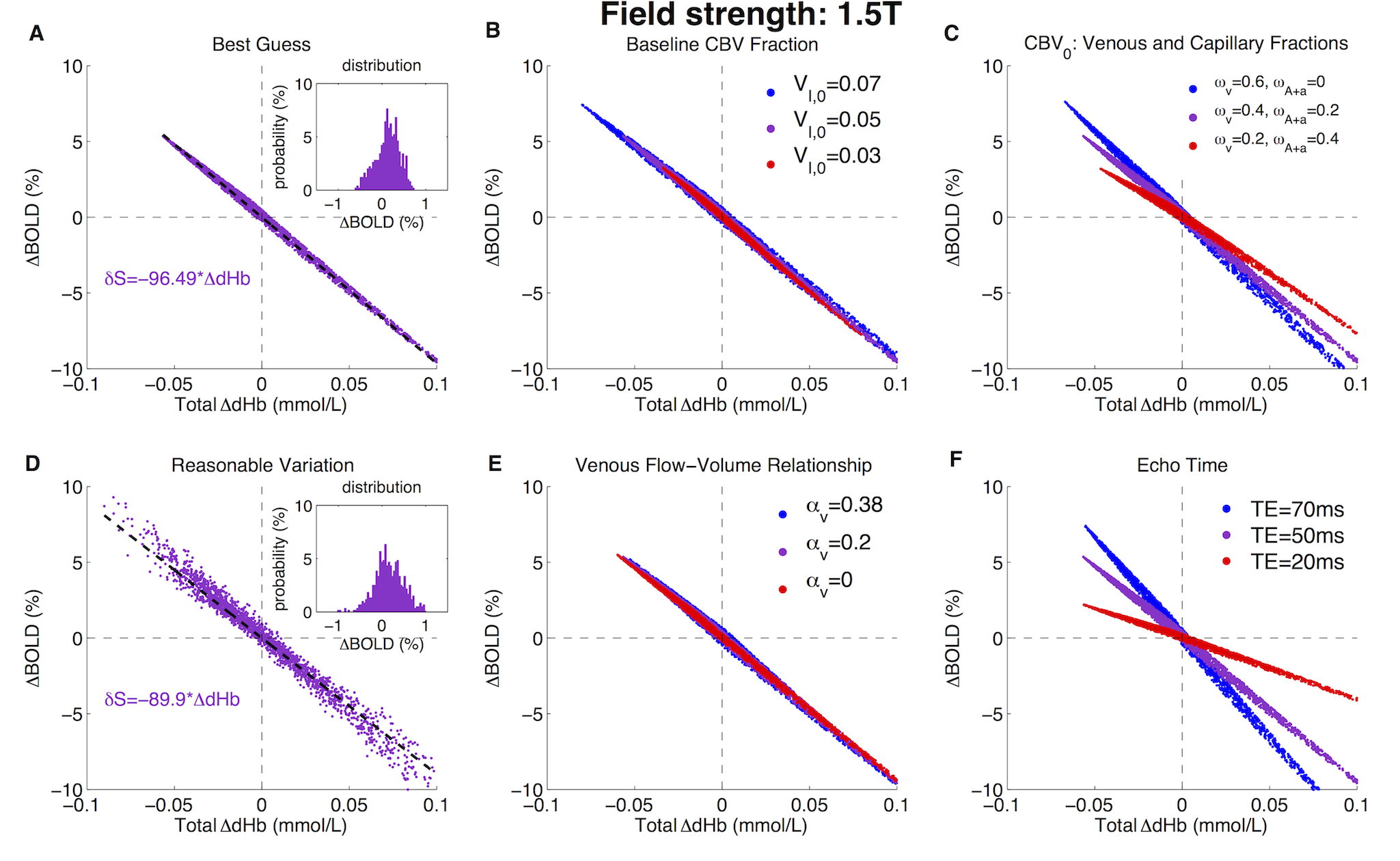

Supplement: Figure S2 — Relationship between the BOLD signal change and the total at 1.5T. Scatter plots were produced by independently varying ΔCBF and ΔCMRO2 as in Figure S1. (A) For the best guess of physiology, the relationship between the BOLD signal and is linear, but again there is a finite width to the curve. For ΔBOLD between −3% and 3%, a fit to this line gives ΔBOLD(%) = −96*. The inset is a histogram of ΔBOLD probability distribution around 0±0.025 mg/mL is similar to that at 3T As expected, the BOLD signal shows weaker dependence on the change in dHb content than at 3T (B–F). (TIF) [file pone.0068122.s002.tif]

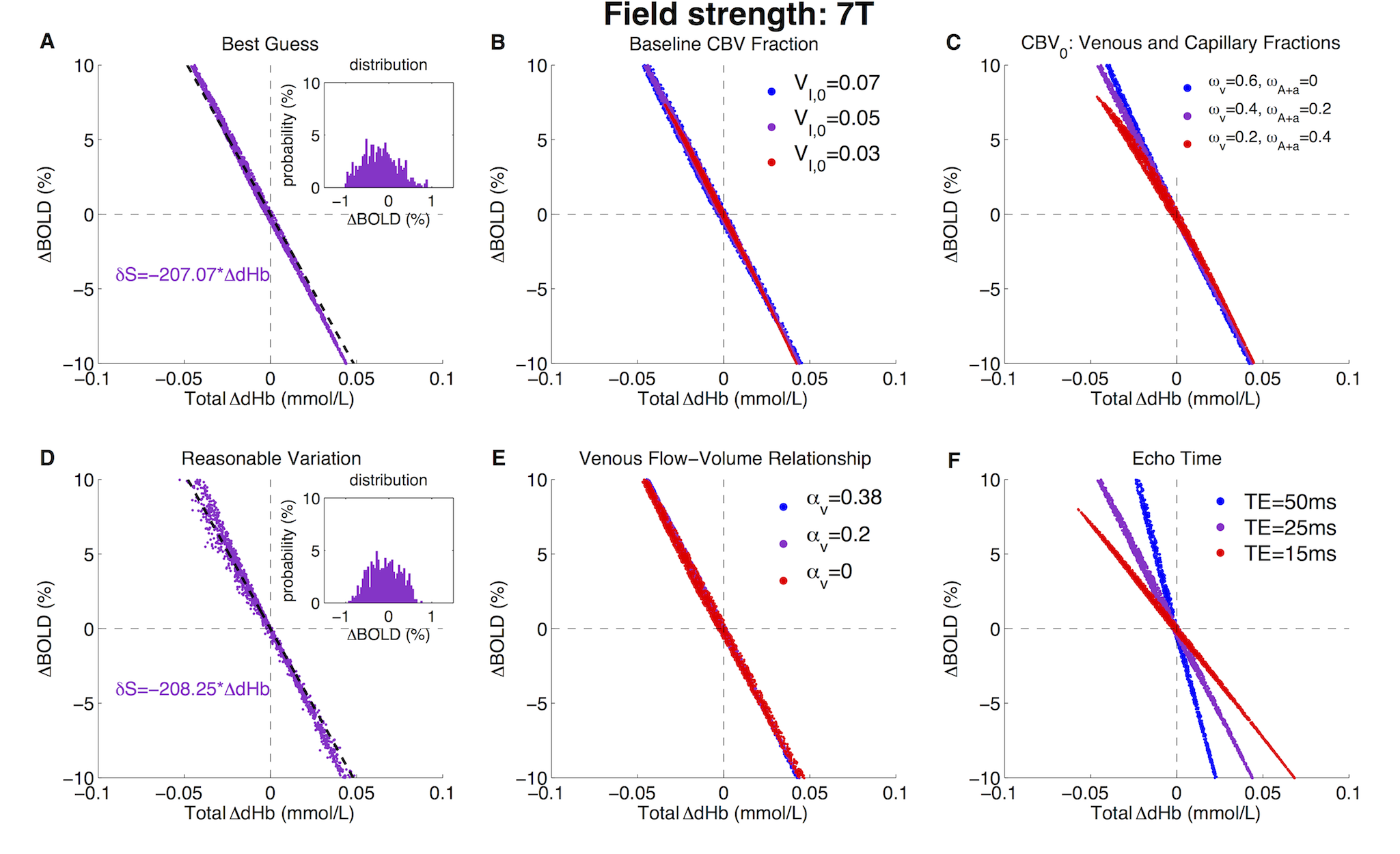

Supplement: Figure S3 — Relationship between the BOLD signal change and the total at 7T. Scatter plots were produced by independently varying ΔCBF and ΔCMRO2 as in Figure S1. (A) For the best guess of physiology, the relationship between the BOLD signal and is linear with a tighter distribution than at 3T or 7T. For ΔBOLD between −3% and 3%, a fit to this data gives ΔBOLD(%) = −207*. As expected, the BOLD signal shows stronger dependence on the change in dHb content than at 3T (B–F). (TIF) [file pone.0068122.s003.tif]

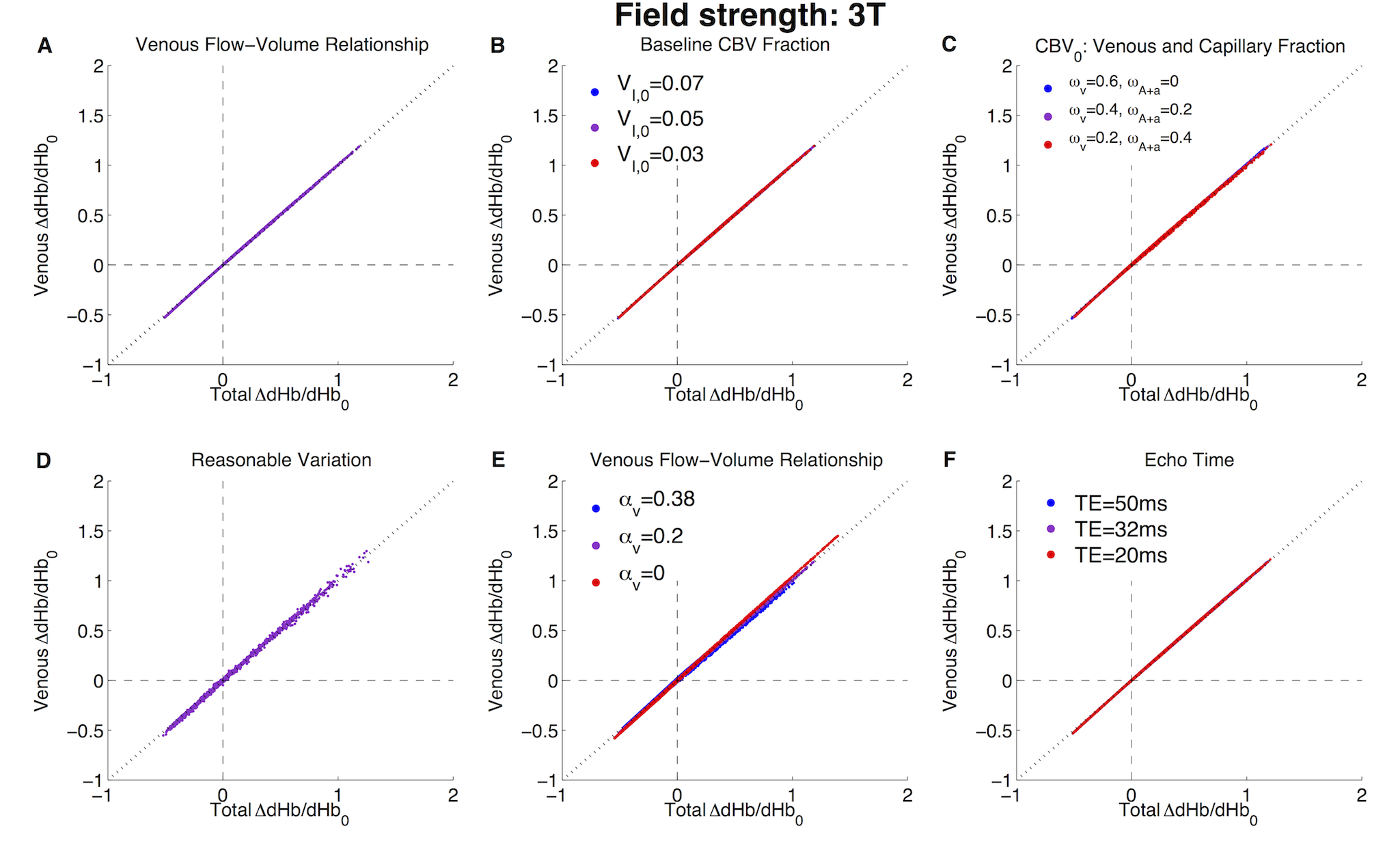

Supplement: Figure S4 — Relationship between the normalized venous change and normalized total change in dHb contents. Scatter plots were produced as in Figure S1 by independently varying ΔCBF (−50% to 80%) and ΔCMRO2 (−30% to 50%) within the specified ranges. Purple curves are identical in all subplots with the exception of (D) and represent the best guess physiological case (Tables 1 and 2). (D) Combined variation of the parameters within the reasonable ranges (Tables 1 and 2). The only physiological variable that created a slight deviation from the identity line is the venous flow-volume relationship expressed as αv (E). (TIF) [file pone.0068122.s004.tif]

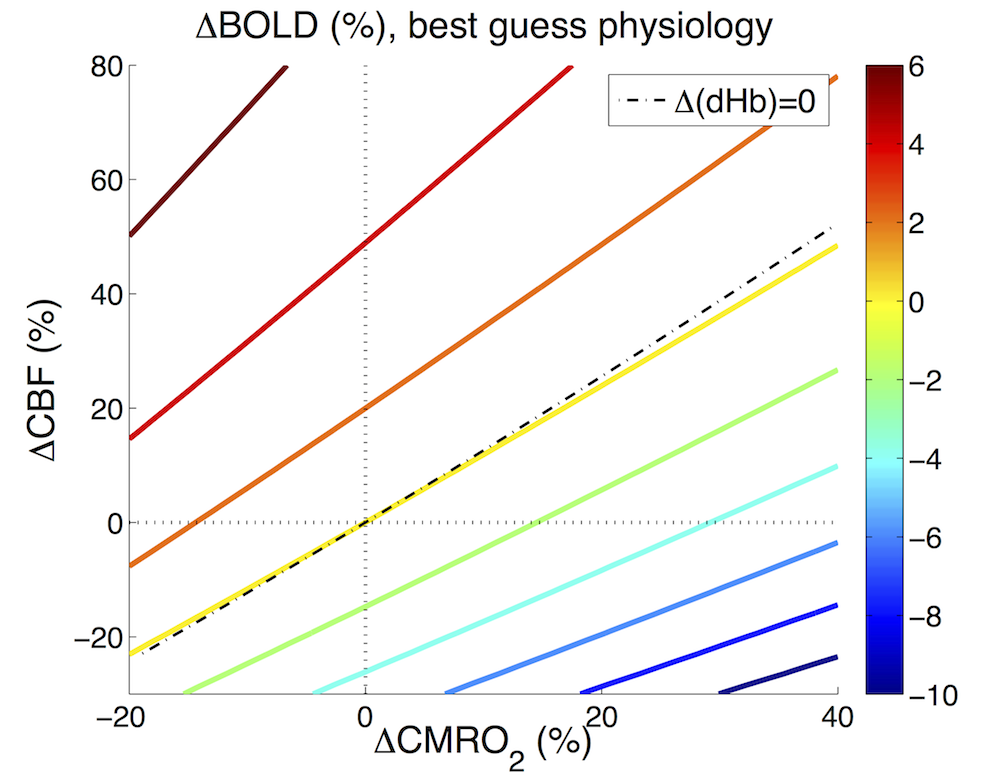

Supplement: Figure S5 — Comparing zero BOLD response to zero change in total dHb content. This plot of the BOLD response as a function of changes in CBF and CMRO2 was generated using our best guess of the physiological inputs to the DBM model at 3T (Tables 1 and 2). The color scale represents the BOLD signal as a percent change. The dot-dash line represents while the solid orange line represents ΔBOLD = 0%. For positive changes in CBF and CMRO2, is shown to be associated with a small positive BOLD signal. This is due to the intravascular effects of dHb: although the increase in CBV and decrease in dHb concentration combine to produce no change in total dHb content and no change in the extravascular signal, the intravascular signal decay rate decreases due to the decrease in dHb concentration. (TIF) [file pone.0068122.s005.tif]

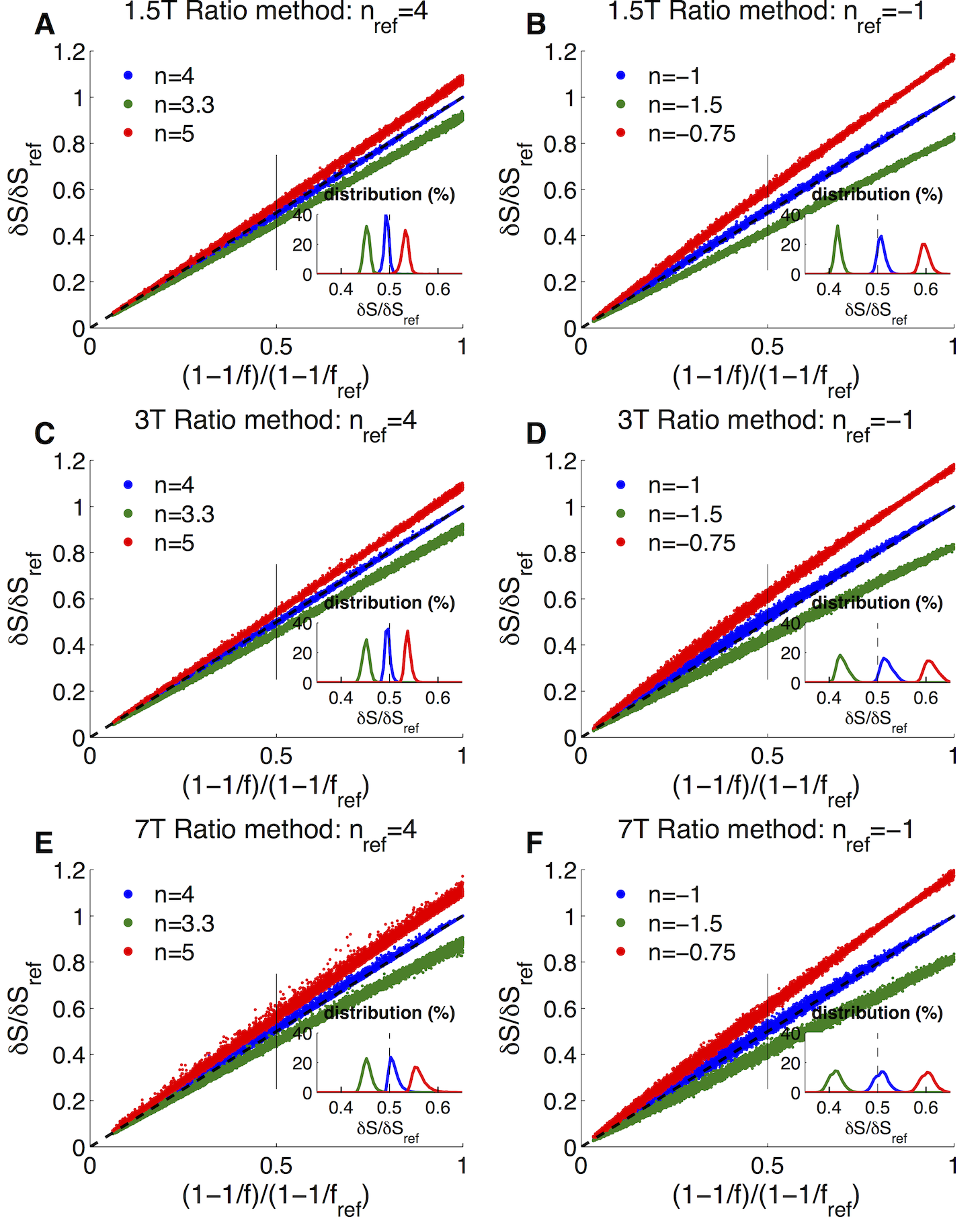

Supplement: Figure S6 — The ratio method for analysis of combined BOLD (δS) and CBF data: effects of different n . The DBM was used to simulate BOLD data from changes in CBF and set values of n. 10,000 simulations were performed using the ranges for the model inputs noted in Tables 1 and 2. The data was compared to a reference of nref = −1 or nref = 4 at B0 = 1.5T, 3T and 7T. Inset histograms show the distribution of δS ratios for a CBF ratio of 0.5. (A,C,E) For nref = 4 at 1.5T (A) and 3T (C), the ratio method appears to work well, although the data is slightly more difficult to distinguish, which is expected due to the decreased sensitivity of the BOLD signal to n at higher values of n. At 7T (E), the approach is again biased when nx = nref. At all three field strengths, the ratio method separates the data well for nref = −1, although there is bias in the nx = nref data. (B,D,F). (TIF) [file pone.0068122.s006.tif]
