# Supplementary material for: A New Functional MRI Approach for Investigating Modulations of Brain Oxygen Metabolism
Source: PLoS One. 2013 Jun 27;8(6):e68122. doi: 10.1371/journal.pone.0068122 (PMC3694916; doi:10.1371/journal.pone.0068122)
Supplement: Appendix S1 — Derivation of the new model. (DOC) [file pone.0068122.s007.doc]

**Appendix A**

*Derivation of the new model*

Both the heuristic model and the Davis model are highly simplified mathematical expressions trying to capture a complex phenomenon. The main text describes tests of the accuracy of these simpler models against the DBM, which reflects our current knowledge of the BOLD signal and is the bottom-line test of the simpler models. Nevertheless, it is important to consider the basic ideas that motivated the proposed heuristic model, which can be derived from two assumptions. Neither of these assumptions is strictly true, and the DBM is used here to test each of these assumptions as the parameters in Tables 1 and 2 are varied.

*Assumption 1*: The fractional BOLD signal change is directly proportional to the absolute change in total deoxyhemoglobin (dHb) content in a voxel with slope *m*:

Here, $\left\langle\text{dHb}\right\rangle$ (“dHb content”) is total dHb per unit voxel volume and is dependent on both hemoglobin oxygen saturation and blood volume. $\left\langle\text{dHb}\right\rangle_{0}$ is the dHb content in the baseline state. For our best guess of the physiology (Tables 1 and 2), this value is 0.11 mmol of dHb per liter of tissue volume assuming a hemoglobin monomer molecular weight of 16.1 kDa. In the seminal work of Ogawa and colleagues , they used Monte Carlo simulations to show that this assumption is true for the extravascular signal change due to larger vessels for which diffusion is not important. Here using the detailed model, we tested whether this is still a reasonably accurate assumption when non-linear effects due to intravascular signal changes and extravascular signal changes around capillaries are incorporated; for 10,000 random combinations of %ΔCBF (-50% to 80%) and %ΔCMRO_2_ (-30% to 50%), we examined the relationship between BOLD and using our best guess of the physiology while also systematically varying these parameters as defined in Tables 1 and 2 (Supporting Figure S1). We looked at the effects of varying baseline CBV fraction (*V*_0_), the fraction of this CBV considered to be venous (ω*_v_*), the coefficient relating venous CBV to CBF (α*_v_*) and echo time (TE). We also examined whether a reasonable amount of combined variation in these parameters as defined in Tables 1 and 2 would have a different effect (Supporting Figure S1, panel D). Supporting Figures S2 and S3 show these simulations for 1.5T and 7T. The linearity of all these results suggests that Eq. (A1) is a reasonable assumption. This leads to the BOLD equation:

Note, though, that the curves in Supporting Figure S1, while supporting an overall linear variation, have a certain thickness to them, indicating the limitations of this assumption. We consider the source of this effect below. In addition to those input parameters to the detailed model noted, we examined the others listed in Table 1; these results are not shown but are consistent with the first assumption.

*Assumption 2*: The fractional change in tissue concentration of total dHb is equal to the fractional change of venous dHb:

Tests of this assumption using the DBM (in parallel to its use in producing Figure S1) are shown (Supporting Figure S4). The data falling along the line of identity suggests that this assumption is accurate as well. We also examined the effect of varying the other parameters listed in Tables 1 and 2 (results not shown) without finding any noticeable effect on this relationship.

The first assumption defines the basic relationship between the BOLD signal and dHb, and the second assumption provides the link to the physiological parameters. We define *f*, *v*, and *r* respectively as CBF, venous CBV and CMRO_2_ normalized to their baseline state values. Also *E* and *E_0_* are the oxygen extraction fractions in the active and baseline states such that *E*=dHb/Hb. Since in most cases *E/E_0_ = r/f* (from the definition of CMRO_2_ with the notable exception of hyperoxia), the normalized change in venous dHb is:

Combining equations [A2-A4], the BOLD equation becomes:

We posit that the normalized change in CMRO_2_ (ΔR/R_0_) and the normalized change in CBV (ΔV/V_0_) are small enough to justify first order approximation while the normalized change in CBF (ΔF/F_0_) is too large for such an approximation:

Defining the coupling parameter *n=*(*f-*1)/(*r-*1) such that and substituting for the approximation , which is the linearization of leads to the following, which can be rearranged:

$$\begin{matrix} 1-\frac{v\cdot r}{f}=1-\frac{1}{f}\left(1+\alpha_{v}\frac{\Delta F}{F_{0}}+\frac{1}{n}\cdot\frac{\Delta F}{F_{0}}\right) \\ \text{ }\approx\frac{\left(1+\Delta F/F_{0}\right)F_{0}}{\left(1+\Delta F/F_{0}\right)F_{0}}-\frac{F_{0}}{\left(1+\Delta F/F_{0}\right)F_{0}}-\frac{\alpha_{v}\cdot\Delta F}{\left(1+\Delta F/F_{0}\right)F_{0}}-\frac{\Delta F}{n\left(1+\Delta F/F_{0}\right)F_{0}} \\ \text{ }\approx\frac{\Delta F/F_{0}}{1+\Delta F/F_{0}}-\frac{\alpha_{v}\cdot\Delta F/F_{0}}{1+\Delta F/F_{0}}-\frac{\Delta F/F_{0}}{n\left(1+\Delta F/F_{0}\right)} \\ \text{ }\approx\left(\frac{1+\Delta F/F_{0}}{1+\Delta F/F_{0}}-\frac{1}{1+\Delta F/F_{0}}\right)\left(1-\alpha_{v}-1/n\right) \\ \text{ }\approx\left(1-1/f\right)\left(1-\alpha_{v}-1/n\right) \end{matrix}$$

The new model of the BOLD signal is therefore:

As noted above, the primary source of error in this model likely arises from the thickness of the lines in Supporting Figure S1. While the overall behavior of the curves is linear, the width of the curves contradicts the assumption that no change in produces no change in the BOLD signal. Specifically, a non-zero BOLD signal may occur even if there is no change in dHb content when an increase in CBF both decreases the blood concentration of dHb and also increases venous CBV. This would result in no extravascular signal change, but the decrease in dHb blood concentration would still produce a positive intravascular BOLD signal. Additionally there is exchange of blood and tissue volumes, which contributes to the signal change. For these reasons, a measured BOLD signal change could occur even when there is no change in total dHb content. The magnitude of this effect at 3T is illustrated (Supporting Figure S5) by showing curves of constant BOLD signal produced by the DBM in the plane of CBF and CMRO_2_ changes with the best guess of the values for the physiological variables (Tables 1 and 2). Plotted with the curves of the BOLD signal is the curve of constant total dHb content, which departs from the null line of the BOLD signal as the CBF and CMRO_2_ changes increase.
