# Supplementary material for: A New Functional MRI Approach for Investigating Modulations of Brain Oxygen Metabolism
Source: PLoS One. 2013 Jun 27;8(6):e68122. doi: 10.1371/journal.pone.0068122 (PMC3694916; doi:10.1371/journal.pone.0068122)
Supplement: Appendix S2 — Limits of the simple models. (DOC) [file pone.0068122.s008.doc]

**Appendix B: Limits of the simple models**

*Maximizing the BOLD signal through elimination of dHb*

In order to maximize the BOLD signal in a particular subject, all dHb present in the baseline state must be eliminated. This can occur through increases in CBF or decreases in CMRO_2_. These limits of the simple models are examined in order to elucidate their relationship to the maximum BOLD signal. The limits of the Davis model are straightforward. In the case of an infinite CBF change, the term goes to zero:

When CMRO_2_ approaches zero (*r*=0), the same solution is found such that in both instances the Davis model scaling parameter is equivalent to the maximum BOLD signal.

The heuristic model is also straightforward for the case of an infinite CBF change as both the 1/*f* and 1/*n* terms go to zero:

In the case that CMRO_2_ approaches zero, the solution is slightly more complicated and depends on CBF, however if ΔCBF is small the limit simply becomes *A*:

*The limit of the heuristic and Davis* *models as ΔCBF approaches zero*

Starting with the Davis model and assuming changes in CBF and CMRO_2_ are small, substitutions can be made for *f*🡪1 and *n*:

Starting with the heuristic model, substitutions can be made for *f* and *n* as follows:

$$BOLD\left(\%\right)=A\left(1-\frac{F_{0}}{F_{0}+\Delta F}\right)\left(1-\alpha_{v}-\frac{F_{0}\cdot\Delta R}{R_{0}\cdot\Delta F}\right)$$

For small changes in flow, we can find the limit of the flow term using a first order approximation since the inverse of 1+x where x is a small number is 1-x. Additionally, a number divided by Δ*F* as this term approaches zero will go to infinity overwhelming the finite 1-α_v_ term:

$$\begin{matrix} BOLD\left(\%\right)=A\left(1-\frac{F_{0}}{F_{0}+\left(1+\Delta F/F_{0}\right)}\right)\left(1-\alpha_{v}-\frac{F_{0}\cdot\Delta R}{R_{0}\cdot\Delta F}\right) \\ \text{ }=A\left[1-\left(1-\Delta F/F_{0}\right)\right]\left(-\frac{F_{0}\cdot\Delta R}{R_{0}\cdot\Delta F}\right) \end{matrix}$$

Solving for the BOLD signal leaves the following relationship:

$$BOLD\left(\%\right)=-A\frac{\Delta R}{R_{0}}$$
